# Experimental Immunogenicity and Multi-Region Field Performance of a Briquette-Formulated Oral Rabies Vaccine Bait in Kazakhstan

**DOI:** 10.3390/biology15161374

**Published:** 2026-08-12

**Authors:** Dariya Toktyrova, Ruslan Abitayev, Abdurakhman Ussembay, Zhanat Amanova, Zhanna Sametova, Sholpan Turyskeldy, Zhanat Kondibayeva, Alina Kurmasheva, Zhumagali Koshemetov, Gourapura J. Renukaradhya, Aida Kistaubayeva, Dana Mazbayeva, Yerbol Bulatov

**Affiliations:** 1 Research Institute for Biological Safety Problems, National Holding “QazBioPharm”, Gvardeyskiy 080409, Kazakhstan; r.abitaev@biosafety.kz (R.A.); a.ussenbay@biosafety.kz (A.U.); zh.amanova@biosafety.kz (Z.A.); zh.sametova@biosafety.kz (Z.S.); sh.smankizi@biosafety.kz (S.T.); zh.kondybaeva@biosafety.kz (Z.K.); a.kurmasheva@biosafety.kz (A.K.); zh.koshemetov@biosafety.kz (Z.K.); d.mazbayeva@biosafety.kz (D.M.); 2 Faculty of Biology and Biotechnology, Department of Biotechnology, Al-Farabi Kazakh National University, Almaty 050040, Kazakhstan; aida.kistaubaeva@kaznu.edu.kz; 3 Animal Science Building, Department of Animal Sciences, The Ohio State University, 2029 Fyffe Road, Columbus, OH 43210, USA; gourapura.1@osu.edu

**Keywords:** rabies, oral rabies vaccination, wildlife reservoirs, oral vaccine bait, ecological heterogeneity, bait uptake, One Health

## Abstract

Wildlife reservoirs sustain rabies transmission despite vaccination of domestic animals, highlighting the need for oral rabies vaccination strategies adapted to contrasting ecological settings. We conducted a pilot multi-region field evaluation of a locally developed briquette-formulated oral rabies vaccine bait at purposively selected sites in four regions of Kazakhstan during 2025–2026. Overall, 9256 baits were deployed, including 4556 placed manually and 4700 distributed by aircraft. Among the manually monitored baits, apparent uptake was 57.2% (2604/4556) and ranged from 11.3% to 75.3% among region-campaign groups. Recovered baits retained their structural integrity following a controlled aerial test drop. Tetracycline fluorescence was detected in seven of ten free-ranging foxes, and six had rabies virus-neutralising antibody titres of ≥0.5 IU/mL. Following controlled oral administration of a single bait, all six initially seronegative captive red foxes and four of five captive raccoons met the predefined seroconversion criterion. These findings support the operational field feasibility of the briquette-formulated bait across contrasting selected landscapes and demonstrate oral immunogenicity under controlled conditions. The observed variation in apparent uptake indicates that bait distribution strategies should be adapted to local wildlife ecology and deployment conditions.

## Simple Summary

Rabies continues to circulate in wild animals and can spread to livestock, pets, and people. Oral rabies vaccination is used to control the disease in wildlife by distributing edible baits containing a vaccine. This study evaluated a locally developed briquette-shaped rabies vaccine bait in selected landscapes of Kazakhstan. During three field campaigns, 9256 baits were distributed manually or from an aircraft. Among the manually monitored baits, approximately 57% were consumed or damaged by animals, although the proportion varied considerably among regions and campaigns. Camera traps recorded red foxes, corsac foxes, golden jackals, and several non-target species interacting with the baits. Recovered baits remained physically intact after a controlled aircraft drop. Evidence of bait exposure was detected in seven of ten free-ranging foxes, and six had measurable antibodies against rabies. In a controlled study, a single bait produced an antibody response in all six captive red foxes and four of five raccoons. These findings support further development of the bait for wildlife rabies control and show that distribution methods should be adapted to local animal species, habitats and activity patterns.

## 1. Introduction

Rabies remains one of the most important zoonotic infections, characterised by an almost invariably fatal outcome once clinical signs develop and by a substantial public health, veterinary, and socio-economic burden. The highest disease burden occurs in countries of Asia and Africa, where rabies causes an estimated 59,000 human deaths annually [1]. Accordingly, the global strategy for rabies control aims to achieve zero human deaths from dog-mediated rabies by 2030 through coordinated vaccination, surveillance, access to post-exposure prophylaxis, and One Health collaboration [2]. Although current international programmes primarily focus on dog-mediated transmission, rabies virus circulation in wildlife populations remains an important source of epizootic risk. Wild carnivore reservoirs can maintain sylvatic transmission cycles and facilitate spill-over to domestic animals, livestock, and humans, particularly in regions where wildlife and domestic animal populations frequently interact [3,4,5].

Oral rabies vaccination (ORV) of wild carnivores is one of the most effective approaches for controlling rabies in wildlife reservoirs. This strategy has been widely implemented in Europe and North America and has contributed to a substantial reduction, and in some areas, local interruption of rabies virus circulation in wildlife populations [6]. ORV programmes targeting red foxes, raccoons, skunks, grey foxes, coyotes, and selected jackal populations are particularly well documented [7]. The principal advantage of ORV is that it enables vaccination of free-ranging animals without direct capture and handling through the spatial distribution of edible baits. Although vaccine safety and immunogenicity are essential prerequisites, the field effectiveness of oral rabies vaccination programmes ultimately depends on successful vaccine delivery to target animals. This requires that vaccine baits are encountered, accepted, and consumed, and that the vaccine sachet is ruptured to release the vaccine into the oral cavity of the target animal [8]. Bait composition and attractiveness, physical and biological stability, distribution density, delivery method, season, landscape structure, natural food availability, and species-specific feeding behaviour may therefore influence vaccination coverage under field conditions.

Most operational evidence supporting wildlife ORV has been generated in Europe and North America, whereas comparable field data remain limited for Central Asia. Established bait formulations and distribution strategies cannot necessarily be assumed to perform uniformly in regions with different reservoir communities, climatic regimes, landscape structures and seasonal patterns of food availability. This knowledge gap is particularly relevant to Kazakhstan, which is characterised by a vast territory and pronounced ecological heterogeneity, including steppe, semi-desert, desert, riparian, foothill and mountain landscapes [9]. These ecological zones differ in climate, vegetation, wildlife community composition, natural food availability and the distribution and activity patterns of wild carnivores, all of which may influence bait accessibility, persistence, attractiveness and consumption by target species. Although oral rabies vaccination has been successfully implemented in Europe and North America, rabies remains epizootic in Kazakhstan. According to national surveillance data, 985 animal rabies cases were recorded between 2013 and 2025 [10]. In addition, 51 human rabies deaths were reported in Kazakhstan during 2010–2020, underscoring the continuing public health importance of the disease [11]. Despite preventive vaccination of domestic animals and measures targeting stray animal populations, several regions remain epizootically unfavourable [10]. The persistence of rabies in livestock and domestic animals is consistent with epidemiological connectivity between sylvatic and domestic transmission cycles, in which wild carnivores may act as reservoir or bridge hosts. Consequently, implementation of ORV in Kazakhstan requires field evidence generated under the ecological and climatic conditions in which the vaccine bait is intended to be used.

The principal wild carnivore species relevant to ORV in Kazakhstan include the red fox (*Vulpes vulpes*), corsac fox (*Vulpes corsac*), and golden jackal (*Canis aureus*). These species differ in geographical distribution, habitat use, feeding ecology, movement behaviour, and seasonal activity, suggesting that a uniform baiting strategy may not achieve comparable uptake across contrasting ecological zones. International experience confirms that wildlife vaccination programmes require adaptation to local ecological and operational conditions [12,13,14]. For Kazakhstan, such adaptation requires consideration of the epizootic situation, target species distribution and activity, campaign timing, bait density, microhabitat selection and the feasibility of manual or aerial distribution. However, these factors have not previously been evaluated together with field bait uptake, camera-trap observations, biomarker-confirmed bait exposure and serological findings in free-ranging target animals under the contrasting ecological conditions of Kazakhstan.

Before field deployment, the briquette-formulated vaccine bait underwent laboratory and preclinical evaluation. Previous studies demonstrated bait acceptance, clinical tolerability, immunogenicity and protective efficacy in dogs and foxes [15,16]. However, these controlled investigations could not evaluate bait performance, wildlife interactions or vaccine-associated immunological responses under heterogeneous natural conditions, thereby providing the rationale for the present field study.

We hypothesised that apparent oral rabies vaccine bait uptake would vary among region-campaign groups and that this variation would be associated, at least in part, with differences in local target-canid activity and environmental conditions. The aim of this pilot study was therefore to conduct a prospective multi-region field evaluation of a briquette-formulated oral rabies vaccine bait intended for wild carnivores under contrasting ecological conditions in Kazakhstan. Specifically, we evaluated the operational feasibility of manual and aerial bait deployment, variation in apparent bait uptake among region-campaign groups, physical bait integrity following aerial release, interactions of target and non-target species with vaccine baits, tetracycline-confirmed bait exposure, and post-campaign rabies virus-neutralising antibody (RVNA) responses in free-ranging foxes. Supporting controlled observations assessed RVNA responses following oral bait administration in captive animals. Together, these complementary assessments were designed to provide an integrated evaluation of the field performance and immunogenic potential of the briquette-formulated oral rabies vaccine bait under diverse ecological conditions.

## 2. Materials and Methods

### 2.1. Vaccine Bait Formulation

A briquette-formulated oral rabies vaccine bait was used as the intervention evaluated in this field implementation study. The edible bait matrix was manufactured using fish meal as an attractant and microcrystalline cellulose (MCC; Hebei Yibang Building Materials Co., Ltd., Jinzhou, China) as a matrix-forming component, and briquetting was performed using a dedicated hydraulic press line (Zhengzhou Taicheng Mining Machinery Co., Ltd., Zhengzhou, China). Each bait measured 4.2 × 3.2 × 2.0 cm and weighed 37–40 g. Each bait contained 0.2 g of tetracycline, which was incorporated as a biomarker of bait exposure. A flexible polymer stick-pack (Qingdao Haide Packaging Co., Ltd., Qingdao, China) sachet containing the vaccine suspension was embedded within the edible matrix. Each vaccine sachet contained 2 mL of a suspension of the attenuated rabies virus strain Rabies virus fix/NIIPBB/2024, propagated in BSR cells cultured on BioNOC II macrocarriers (Esco Micro Pte. Ltd., Singapore) in a BelloStage™-3000 bioreactor (Esco Micro Pte. Ltd., Singapore) [17]. The strain was obtained from the Microorganism Collection of the Research Institute for Biological Safety Problems (RIBSP), Gvardeiskiy, Kazakhstan. The vaccine suspension had an infectivity titre of 10^6.75^ TCID_50_/mL. Virus infectivity was determined by endpoint dilution in BSR cells, a clone of the BHK-21 cell line, and titres were calculated using the Reed–Muench method. Detailed information on vaccine production, preclinical safety, immunogenicity and protective efficacy has been reported previously [15,16]. All procedures involving propagation, titration or direct handling of live rabies virus were conducted under BSL-3 conditions by personnel authorised to work with risk-group pathogens.

### 2.2. Study Area and Study Design

The study was designed as a prospective, multi-region field implementation study evaluating ecological and operational variation in the apparent uptake of oral rabies vaccine baits. Field campaigns were conducted in Zhambyl, Turkestan, Kyzylorda, and Akmola Regions of Kazakhstan during spring 2025 (March–April), autumn 2025 (October–November) and spring 2026 (March–May).

**Figure 1:**
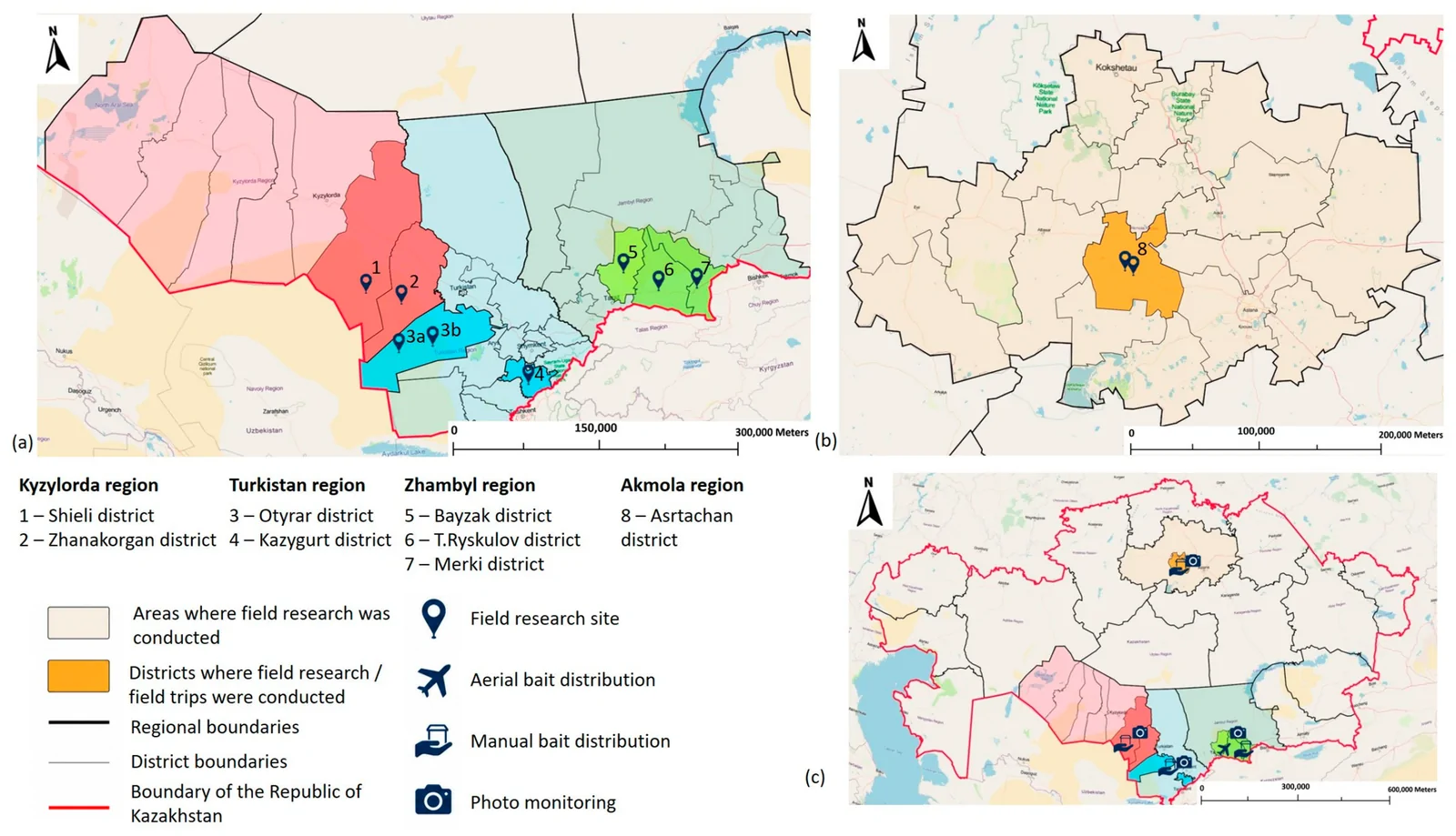
Study regions and operational design of ORV bait deployment in Kazakhstan. (**a**) Study districts in southern Kazakhstan, including Shieli (1) and Zhanakorgan (2) districts in Kyzylorda Region, Otyrar (3) and Kazygurt (4) districts in Turkestan Region, and Bayzak (5), T. Ryskulov (6) and Merke (7) districts in Zhambyl Region. (**b**) Astrakhan District (8) in Akmola Region. (**c**) National overview showing the spatial distribution of the study regions and principal field operations. Coloured polygons indicate the regions and districts in which field activities were conducted. Symbols indicate GPS-referenced field sites, manual bait placement, aerial bait distribution, and camera-trap monitoring. Regional, district, and national boundaries are shown for spatial reference.

The study included eight districts representing steppe, semi-desert, desert, riparian, and foothill environments: Shieli and Zhanakorgan districts in Kyzylorda Region; Otyrar and Kazygurt districts in Turkestan Region; Bayzak, T. Ryskulov, and Merke districts in Zhambyl Region; and Astrakhan District in Akmola Region. The geographical distribution of the study areas and the principal field operations, including manual bait placement, aerial bait distribution, and camera-trap monitoring, are shown in Figure 1.

Study districts were selected purposively on the basis of previous rabies occurrence, evidence of target-canid activity, habitat suitability, accessibility and recommendations from regional wildlife and veterinary authorities. Accordingly, the sampled locations were not intended to constitute a probability sample of the entire territory of Kazakhstan.

A field site was defined as a GPS-referenced location containing a cluster of manually deployed baits within a prespecified local habitat. A site visit was defined as the observation of one field site during one campaign. When the same geographical site was used in more than one campaign, the site retained the same unique site identifier but was assigned a separate site-visit identifier. Site visits were used to organise ecological and spatial records, whereas individual bait outcomes were retained for operational summaries of bait fate. Bait outcomes were also aggregated within site visits to permit site-level description of apparent uptake under shared local environmental conditions.

The primary field outcome was site-level apparent bait uptake, defined as the proportion of manually monitored baits that were completely consumed or partially damaged during the observation period. Secondary outcomes were the descriptive composition and behaviour of target and non-target species recorded by camera traps, tetracycline biomarker detection and rabies virus-neutralising antibody (RVNA) seropositivity in free-ranging foxes, structural integrity of recovered baits following controlled aerial release, and RVNA responses following controlled oral bait administration in captive animals.

### 2.3. Manual Bait Deployment and Site-Level Monitoring

A total of 9256 oral rabies vaccine baits were deployed, including 4556 manually deployed baits and 4700 baits distributed by aircraft. Only manually deployed baits with documented post-deployment inspection were included in the quantitative analysis of apparent bait uptake.

Allocation was based on the estimated abundance and distribution of the principal wild carnivore reservoir species (red foxes, corsac foxes, golden jackals, and wolves), information provided by local hunting management organisations, expected movement patterns of target species, and landscape characteristics. Because this study was designed as a pilot field implementation study, bait allocation was refined between successive campaigns according to operational experience and local ecological conditions rather than being predetermined by statistical power calculations, as the primary objective was to evaluate operational feasibility and bait uptake under field conditions rather than estimate population-level vaccination coverage.

**Figure 2:**
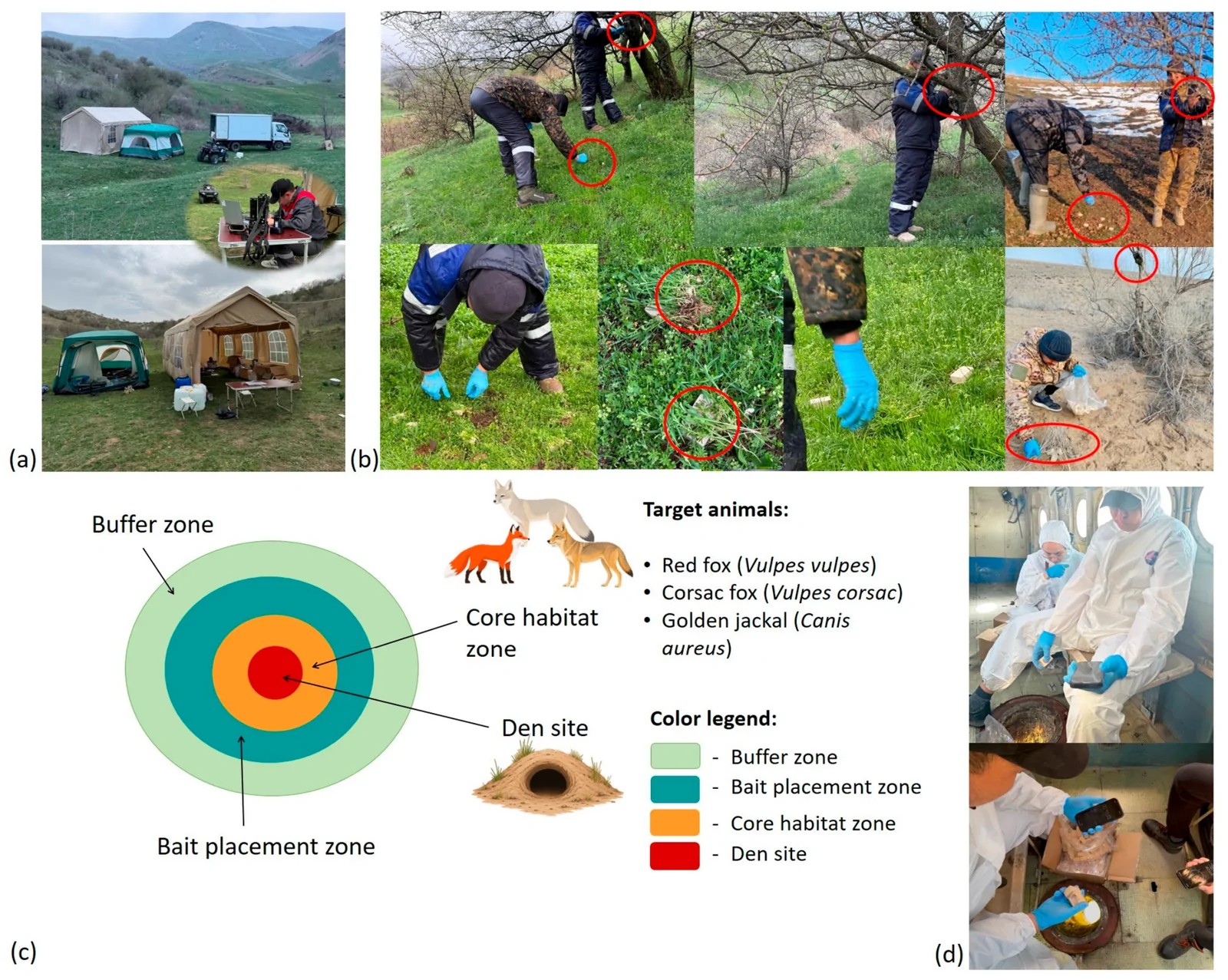
Field implementation of oral rabies vaccine bait deployment. (**a**) Mobile field camp used during field operations, including field registration and preparation of monitoring equipment. (**b**) Manual placement of vaccine baits in selected microhabitats. (**c**) Conceptual arrangement of bait placement around a carnivore den or activity site. (**d**) Aerial distribution of vaccine baits using an Antonov AN-2 aircraft.

Baits were transported under cold-chain conditions at approximately −20 °C and manually deployed at intervals of 30–50 m in microhabitats associated with wild-carnivore activity, including animal trails, denning areas, ravines, water sources, and sites showing recent animal signs (Figure 2). To minimise human visibility and direct sunlight exposure, baits were partially concealed with local vegetation.

Each deployment site was georeferenced using a handheld GPS navigator (GPSMAP 64s; Garmin International, Inc., Olathe, KS, USA). For each site, the geographic location, deployment and inspection times, number of deployed baits, microhabitat characteristics, and bait fate were recorded. At post-deployment inspection, bait fate was classified as complete consumption or removal with evidence of animal interaction, partial consumption or mechanical damage, displacement without visible consumption or damage, intact, or indeterminate. Apparent bait uptake comprised complete consumption or removal with evidence of animal interaction and partial consumption or mechanical damage. Baits classified as displaced, intact or indeterminate were not counted as apparent uptake. All field activities were carried out in cooperation with regional wildlife inspectors of the Ministry of Ecology and Natural Resources of the Republic of Kazakhstan.

### 2.4. Aerial Bait Distribution and Assessment of Post-Release Structural Integrity

Aerial bait distribution was conducted in the Zhambyl, T. Ryskulov, and Merke districts of Zhambyl Region using an Antonov AN-2 light aircraft. Before the principal aerial operation, a controlled test drop was conducted to assess the ability of the briquette matrix and internal vaccine sachet to tolerate the planned release conditions. Baits were released from an altitude of approximately 250–300 m above ground level at a flight speed of 120–130 km/h. The release interval was 10–15 s, with four to five baits released during each drop cycle.

The aircraft flight path was recorded on board using a handheld GPS navigator (GPSMAP 64s; Garmin International, Inc., Olathe, KS, USA). Because exact landing coordinates were not available for every aerially released bait, recorded points were interpreted as flight-route or approximate release locations rather than confirmed bait landing positions.

During the controlled test drop, 100 baits were released and 78 were recovered. Recovered baits were assessed for retention of briquette shape, fragmentation of the edible matrix, integrity of the vaccine sachet, visible leakage, and contamination with soil, water, or other environmental material. Post-release structural integrity was treated as a qualitative, descriptive operational endpoint.

### 2.5. Camera-Trap Assessment of Animal-Bait Interactions

Wild members of the family Canidae considered the principal target species were the red fox (*Vulpes vulpes*), corsac fox (*Vulpes corsac*) and golden jackal (*Canis aureus*). Contacts by non-target wildlife and domestic animals were recorded separately because these interactions could reduce bait availability to target species.

Animal visits and interactions with baits were assessed using 17 BolyGuard BG410-DFP camera traps (Boly Inc., Shenzhen, China). Cameras were installed at a height of 50–80 cm above ground on trees, poles or other stable supports at a distance of approximately 4–6 m from the bait placement site.

For each camera, the installation location, monitoring start and end times, operational duration, and time-stamped animal-bait records were documented. Records were classified as detections of a target wild canid, non-target wild animal, domestic animal or unidentified species. Where visual confirmation was possible, behaviour was classified as investigation, mechanical damage, displacement, partial consumption, complete consumption or removal, or no direct interaction. Because individual animals could not always be distinguished, camera-trap records were used to characterise species occurrence, timing and behaviour rather than abundance or population size.

### 2.6. Assessment of Bait Consumption in Free-Ranging Foxes

Ten free-ranging foxes were captured 21 days after manual bait placement, including five animals in Shieli District, Kyzylorda Region, and five in T. Ryskulov District, Zhambyl Region. Each animal underwent a general clinical examination; however, sex, estimated age class, and body condition score were not recorded.

Before tooth and blood collection, the foxes were chemically immobilised by intramuscular administration of xylazine hydrochloride at 3 mg/kg body weight, corresponding to 0.15 mL/kg (Xyla 2%, 20 mg/mL; Interchemie werken “De Adelaar” B.V., Venray, The Netherlands). Before atraumatic extraction of one first premolar tooth (PM1), regional dental analgesia was provided using 2% lidocaine hydrochloride at 0.2–0.3 mL/kg body weight, corresponding to 4–6 mg/kg (Lidocanit Vet, 20 mg/mL; NITA-FARM LLC, Saratov, Russia). The procedures were performed by a veterinarian in accordance with the approved animal-handling protocol. One first premolar (PM1) tooth was atraumatically extracted from each fox after adequate immobilisation and regional analgesia had been confirmed. The animals were subsequently monitored for bleeding and other immediate complications. The extracted tooth was cleaned of adherent blood and soft tissue, placed in an individually labelled light-protected tube, transported under frozen conditions and stored at −20 °C until laboratory examination.

For tetracycline biomarker detection, non-decalcified longitudinal sections approximately 100–150 µm thick were prepared from each PM1 tooth using a low-speed diamond saw. The sections were mounted on glass microscope slides and examined at ×100 magnification using a Leitz Orthoplan fluorescence microscope equipped for UV excitation (Leitz, Wetzlar, Germany). A tooth was classified as tetracycline-positive when a distinct yellow fluorescent band consistent with tetracycline deposition was observed within the dental tissues.

After tooth collection, blood samples were collected into 6 mL BD Vacutainer SST II Advance serum separator tubes containing a clot activator and gel separator (Becton, Dickinson and Company, Franklin Lakes, NJ, USA) from the saphenous vein of the pelvic limb after disinfection of the venipuncture site with 70% ethanol.

Following completion of the procedures, each fox was monitored until full recovery from chemical immobilisation. Animals were released at their original capture locations only after normal locomotion and the absence of overt procedure-related complications had been confirmed.

### 2.7. Supporting Controlled Oral Administration Study

**Figure 3:**
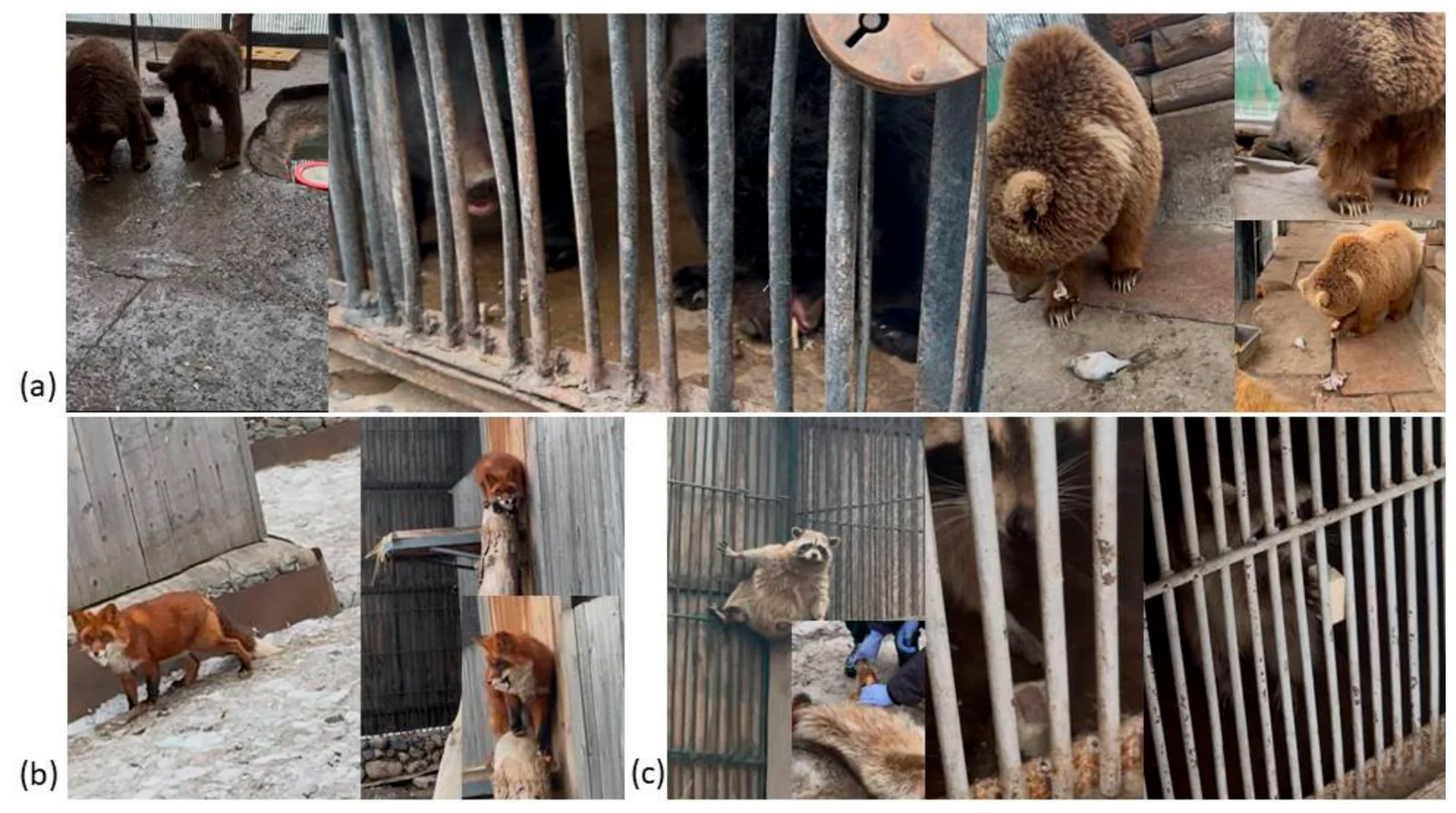
Consumption of oral rabies vaccine baits by animals at Almaty Zoo: (**a**) bears; (**b**) red foxes; and (**c**) raccoons.

A supporting controlled oral administration study was conducted in captive red foxes (*n* = 6), raccoons (*n* = 5) and bears (*n* = 5) housed at Almaty Zoo (Figure 3). All selected animals had no history of previous rabies vaccination. Baseline RVNA seronegativity was confirmed in red foxes and raccoons, whereas baseline serological testing was not performed in bears. This component was included to provide supporting evidence of bait acceptance, oral immunogenicity, and clinical tolerability and was not part of the primary ecological field analysis.

Raccoons (*Procyon lotor*) do not occur in the wild in Kazakhstan and were included only as an additional captive species for assessment of bait acceptance and serological response. Brown bears (*Ursus arctos*) were included as a non-target wildlife species associated with mountain ecosystems in southern and south-eastern Kazakhstan and were evaluated in an exploratory high-dose exposure assessment.

Each fox and raccoon received one vaccine bait under direct observation. Each bear received ten vaccine baits in a single administration to assess bait acceptance and overt clinical tolerability following exposure to an increased bait dose. Complete or partial consumption, rupture of the vaccine sachet, and visible release of the vaccine suspension were recorded using a standardised observation form.

Blood samples were collected from red foxes and raccoons before oral administration and on day 21. Samples were collected into 6 mL BD Vacutainer SST II Advance serum separator tubes. Blood samples were not collected from bears because sampling would have required additional chemical immobilisation. Consequently, bears were included only in the clinical observation component and were not included in the serological analysis. All animals were monitored for 35 days after bait administration for changes in behaviour, appetite, and general activity, as well as for overt neurological or other clinical abnormalities. Animal allocation was not randomised because all eligible animals received the intervention. Laboratory personnel performing the RFFIT were blinded to animal identity.

### 2.8. Determination of Rabies Virus-Neutralising Antibodies

Blood samples were centrifuged at 3000× *g* for 15 min. The separated sera were stored at −20 °C until analysis. The sera were tested for rabies virus-neutralising antibodies (RVNA). Before the assay, serum samples were heat-inactivated at 56 °C for 30 min. Virus-neutralising activity was determined using the rapid fluorescent focus inhibition test (RFFIT) [18] with the BSR cell line, a derivative of BHK-21 cells susceptible to rabies virus infection. The BSR cell line was obtained from the Laboratory of Cell Biotechnology at RIBSP. Cells were cultured in DMEM (Gibco, Thermo Fisher Scientific, Waltham, MA, USA) supplemented with 10% fetal bovine serum (FBS; Capricorn Scientific GmbH, Ebsdorfergrund, Germany) at 37 °C in a humidified atmosphere containing 5% CO_2_. CVS-11 was used as the challenge virus, and FITC-conjugated anti-rabies antibodies (Fujirebio Diagnostics, Inc., Malvern, PA, USA) were used for fluorescent detection. Seroconversion was defined as the detection of an RVNA titre ≥ 0.5 IU/mL on day 21 after vaccination in animals that were seronegative before vaccination.

### 2.9. Statistical Analysis

Statistical analysis was performed using GraphPad Prism version 8.4.3. Bait uptake proportions were calculated for each region and campaign with 95% confidence intervals. RVNA titres after oral administration were compared separately in red foxes and raccoons using the Wilcoxon matched-pairs signed-rank test. Camera-trap observations were analysed descriptively according to animal species, visit time, and bait-interaction behaviour. Differences were considered statistically significant at *p* < 0.05. Cartographic visualisation was performed using ArcGIS Earth version 2.7.1.

### 2.10. Ethical and Regulatory Considerations

This study was conducted in accordance with a protocol approved by the Ethics Committee of the Research Institute for Biological Safety Problems (RIBSP), approval No. 3 dated 3 November 2023. Field activities were authorised by the Committee for Veterinary Control and Supervision of the Ministry of Agriculture of the Republic of Kazakhstan and the Committee for Forestry and Wildlife of the Ministry of Ecology and Natural Resources of the Republic of Kazakhstan, based on permit documents No. 27-03-16/446-KLKhZhM dated 17 March 2025 and No. 27-03-14/1021-KLKhZhM dated 26 February 2026.

All animal procedures were performed in compliance with biosafety requirements and measures aimed at minimising stress and reducing handling time. After examination and full recovery from chemical immobilisation, free-ranging foxes were released at their original capture sites. Personnel involved in handling vaccine baits and wild animals had been vaccinated against rabies and had received biosafety training.

During bait distribution, the risk of accidental contact of humans, domestic animals, and non-target species with vaccine material was taken into account. Protective gloves were used, direct contact with the vaccine suspension was avoided, and any unexpected incidents were documented when they occurred.

## 3. Results

### 3.1. Multi-Region Deployment of Oral Rabies Vaccine Baits

**Figure 4:**
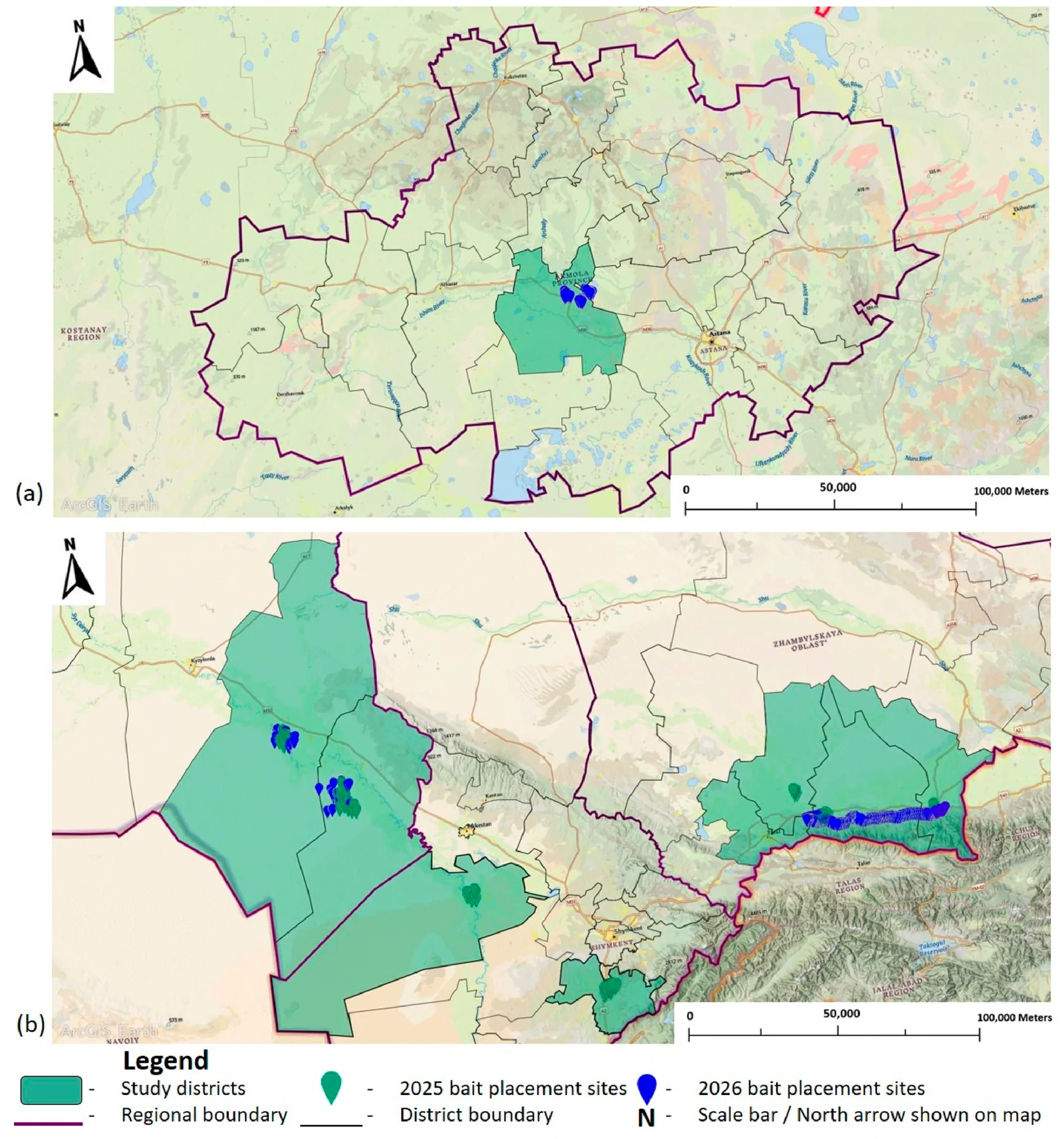
Realised spatial distribution of oral rabies vaccine bait deployment sites during the 2025–2026 field campaigns. (**a**) GPS-referenced deployment sites in Akmola Region. (**b**) Deployment sites in southern Kazakhstan, including Kyzylorda, Turkestan and Zhambyl Regions. Shaded areas indicate the study districts. Green symbols indicate deployment sites used during the 2025 campaigns, and blue symbols indicate sites used during the 2026 campaign. Manual and aerial deployment locations are distinguished by symbol shape. Regional and district boundaries are shown for spatial reference.

Three field campaigns were conducted in spring 2025, autumn 2025 and spring 2026 across Zhambyl, Turkestan, Kyzylorda and Akmola Regions of Kazakhstan. The campaigns covered eight districts representing contrasting steppe, semi-desert, desert, riparian, and foothill environments (Figure 4 and Table 1).

A total of 9256 briquette-formulated oral rabies vaccine baits were deployed during this study. Of these, 4556 baits were placed manually and included in quantitative monitoring of apparent bait uptake, whereas 4700 baits were distributed by aircraft and assessed descriptively for operational deployment and post-release structural integrity. Accordingly, all quantitative estimates of bait uptake reported below were derived exclusively from manually placed and subsequently inspected baits.

Of the 4556 manually deployed baits included in the quantitative analysis, 3056 were deployed during the 2025 field campaigns, whereas the remaining 1500 were deployed during the spring 2026 campaign.

During the 2025 campaigns, 3056 vaccine baits were manually deployed at 104 GPS-referenced sites across seven districts in Zhambyl, Turkestan, and Kyzylorda Regions. The combined field routes covered approximately 100 km. Bait density ranged from approximately 25 to 32 baits/km^2^.

During spring 2026, a further 1500 baits were placed manually. In Kyzylorda Region, 600 baits were distributed at 34 GPS-referenced sites across two districts along approximately 63 km of field routes. In Akmola Region, 600 baits were deployed at 25 sites in Astrakhan District along approximately 23 km of routes. In Zhambyl Region, 300 baits were manually placed at 12 sites, and an additional 4700 baits were distributed by aircraft across Zhambyl, T. Ryskulov, and Merke Districts. The aerial campaign covered approximately 142 km after the exclusion of water bodies and urbanised areas. The aerial distribution route included 212 GPS-referenced flight waypoints.

### 3.2. Aerially Deployed Baits Retained Post-Release Structural Integrity

Aerial delivery of vaccine baits was performed in Zhambyl Region using an Antonov AN-2 light aircraft. The flight route extended from the mountainous areas near the city of Taraz towards the border zone of Merke District and the Teskentogan area.

Baits were released from an altitude of approximately 250–300 m above ground level at a flight speed of 120–130 km/h. The release interval was 10–15 s, with 4–5 baits released during each drop cycle. During the flights, GPS coordinates of the flight route and approximate bait distribution points were recorded.

**Figure 5:**
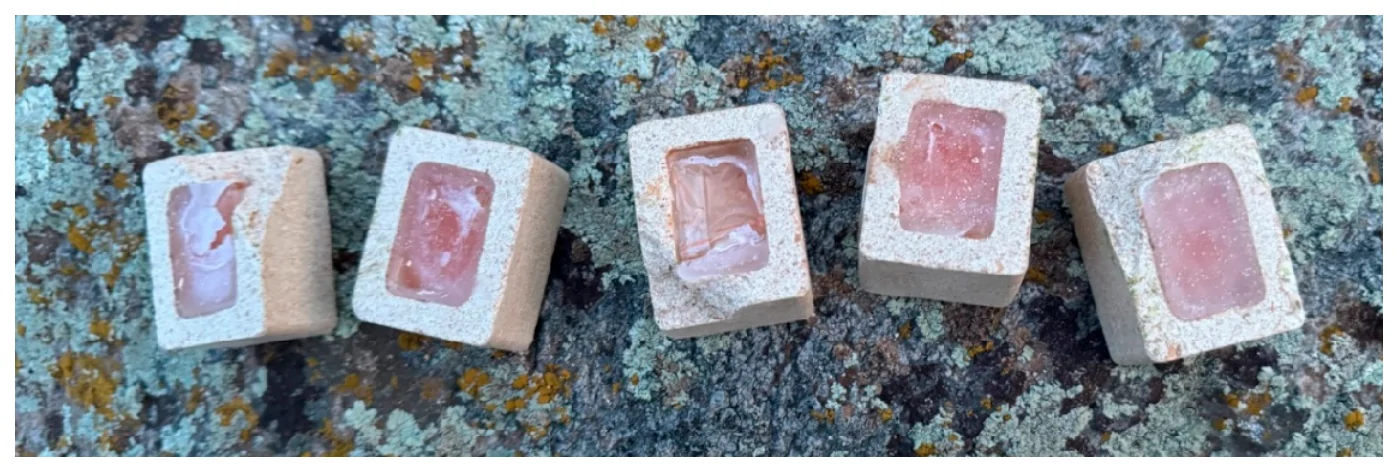
Post-release structural integrity of briquette-formulated oral rabies vaccine baits. Representative baits recovered after a controlled aerial test drop retained the external briquette matrix and intact internal vaccine-containing sachets without visible leakage.

A controlled test drop was performed before the main aerial campaign to assess whether the briquette matrix and internal vaccine-containing sachet remained intact under the planned release conditions. Of the 100 baits released during the controlled test drop, 78 were recovered. The recovered baits retained their briquette shape and contained intact vaccine sachets, with no visible leakage of the vaccine suspension (Figure 5).

These observations provided descriptive evidence that the physical integrity of the baits was retained under the tested aerial release conditions.

### 3.3. Camera Traps’ Documented Interactions by Target and Non-Target Species

Seventeen camera traps were installed at selected bait deployment sites to characterise wildlife occurrence and animal–bait interactions. More than 400 time-stamped detection events were identified. These records were used to describe detected species, timing and bait-interaction behaviours rather than to estimate abundance or the number of individual animals. Recorded behaviour included bait investigation, partial consumption, complete consumption or removal, displacement from the original placement site and no direct interaction.

**Figure 6:**
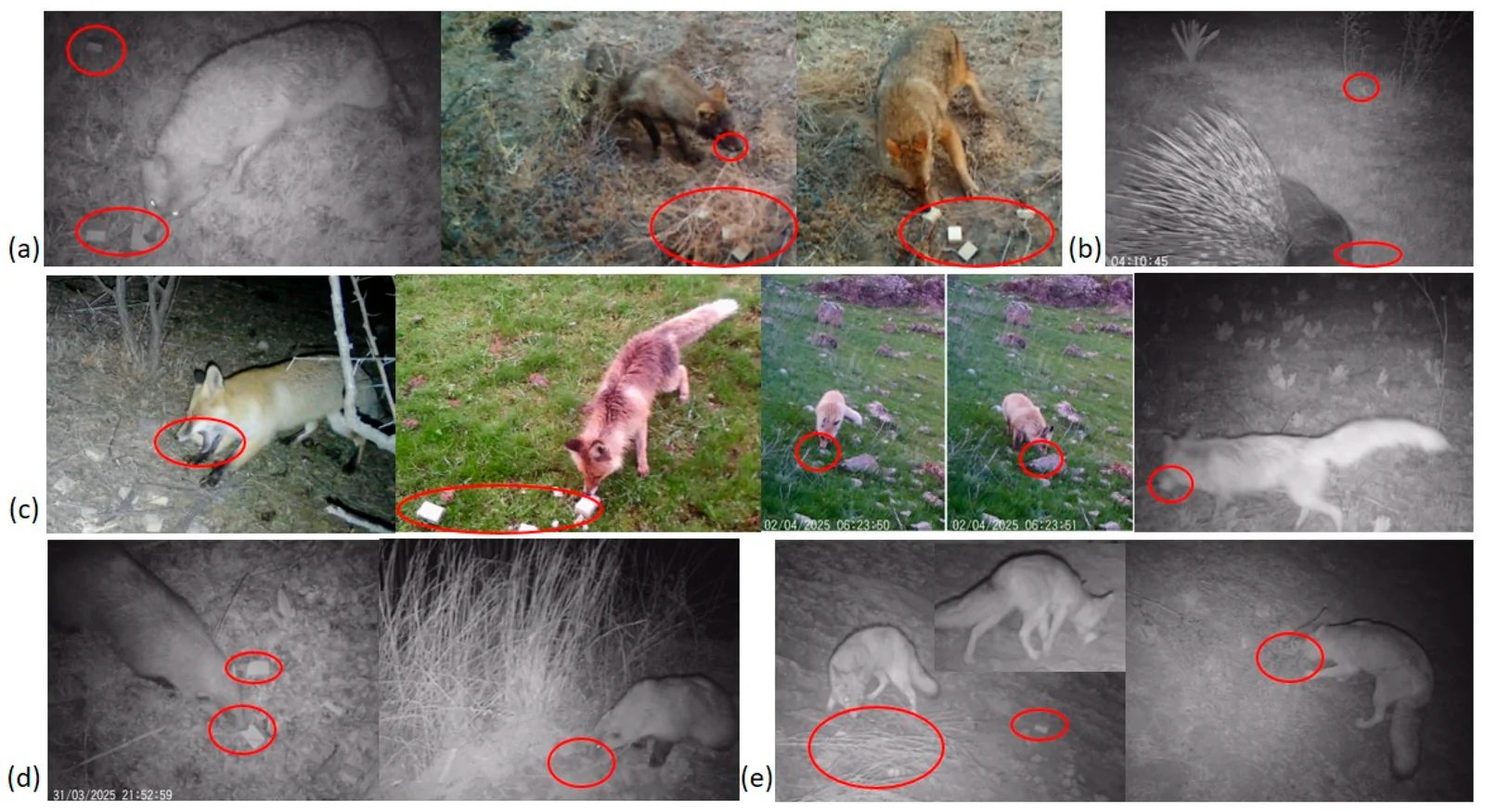
Camera-trap records of target and non-target species interacting with oral rabies vaccine baits. Representative records show (**a**) golden jackals; (**b**) porcupines; (**c**) red foxes; (**d**) badgers; and (**e**) corsac foxes. Red circles indicate the position of the vaccine bait or the site of animal-bait interaction.

Target carnivores recorded at bait sites included red foxes (*Vulpes vulpes*), corsac foxes (*Vulpes corsac*) and golden jackals (*Canis aureus*). Non-target wildlife included porcupines, badgers, wild boars and birds (Figure 6).

The composition of target carnivore detections differed among study regions. Red foxes and golden jackals were commonly observed at bait sites in Kyzylorda, Turkestan and Zhambyl Regions, whereas corsac foxes were the principal target carnivores recorded in Astrakhan District of Akmola Region.

Most detections occurred between 18:00 and 04:00. Red fox detections were predominantly nocturnal, whereas golden jackals were recorded during both nocturnal and daylight periods. Camera-trap observations documented complete bait consumption, partial damage, displacement or removal of baits from their original placement sites, and investigation of baits without visible consumption.

### 3.4. Apparent Bait Uptake Varied Among Region-Campaign Groups

The primary field endpoint was apparent bait uptake, defined as complete bait consumption or partial bait damage identified during post-deployment site inspection. Among 4556 manually placed and monitored vaccine baits, 2604 met this endpoint, corresponding to an overall apparent uptake of 57.2% (exact 95% confidence interval (CI), 55.7–58.6%).

During spring 2025, 459 of 740 monitored baits in Turkestan Region were completely consumed or partially damaged, corresponding to an apparent uptake of 62.0% (95% CI, 58.4–65.5%). In Zhambyl Region, 502 of 740 baits met the endpoint, corresponding to an apparent uptake of 67.8% (95% CI, 64.3–71.2%). Overall, 961 of 1480 monitored baits met the endpoint during the spring 2025 campaign (64.9%).

During autumn 2025, apparent uptake was recorded for 505 of 856 monitored baits in Kyzylorda Region (59.0%; 95% CI, 55.6–62.3%) and 464 of 720 baits in Zhambyl Region (64.4%; 95% CI, 60.8–67.9%). Across the autumn 2025 campaign, 969 of 1576 monitored baits met the endpoint (61.5%).

**Figure 7:**
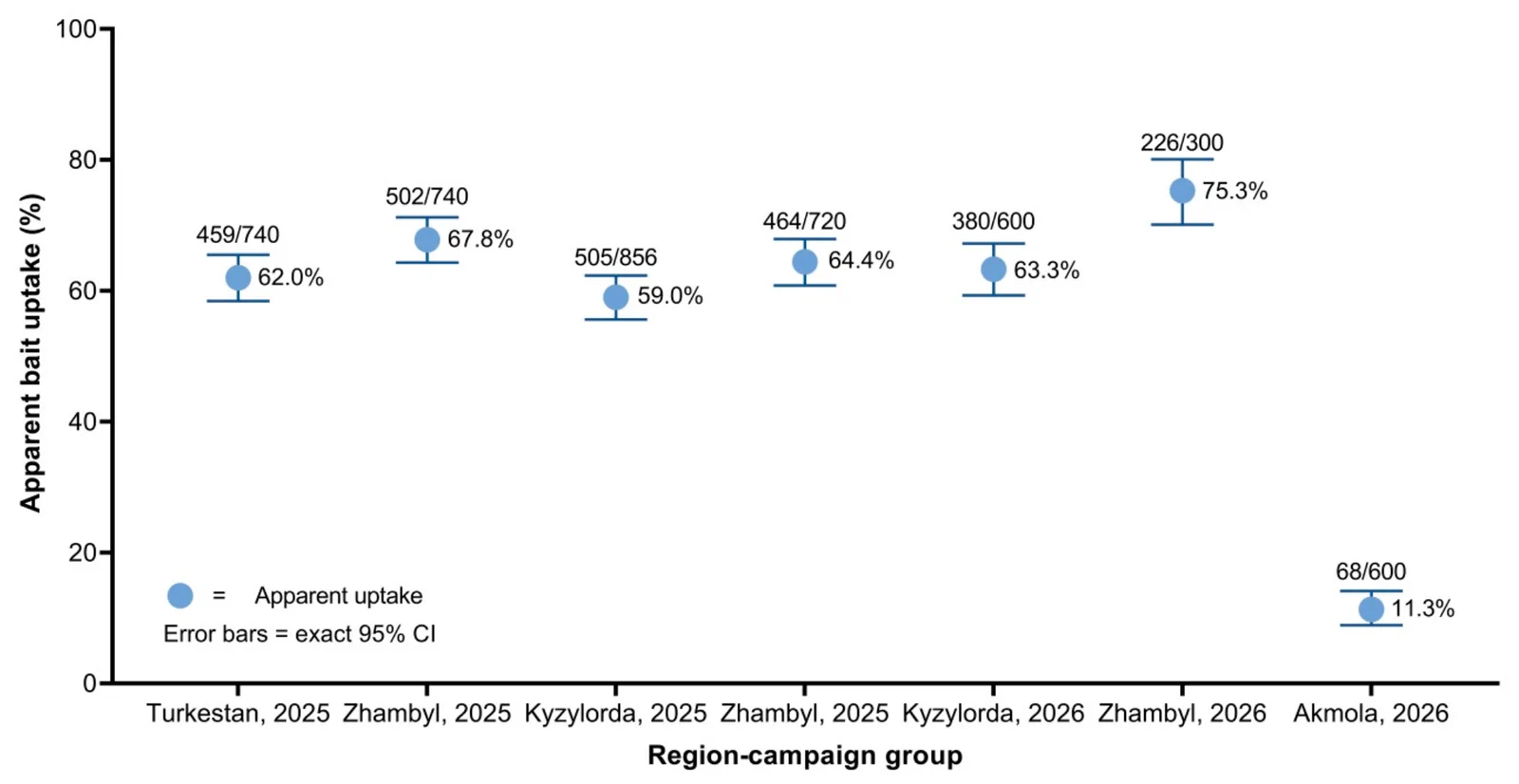
Apparent oral rabies vaccine bait uptake across region-campaign groups. Points indicate the proportions of manually monitored baits that were completely consumed or partially damaged during post-deployment site inspection. Error bars show exact 95% binomial confidence intervals calculated using the Clopper–Pearson method. Labels above the estimates indicate the number of baits meeting the field endpoint divided by the total number monitored in each region-campaign group.

During spring 2026, apparent uptake was recorded for 380 of 600 monitored baits in Kyzylorda Region (63.3%; 95% CI, 59.3–67.2%) and 226 of 300 baits in Zhambyl Region (75.3%; 95% CI, 70.1–80.1%). In Akmola Region, 68 of 600 monitored baits met the endpoint, corresponding to an apparent uptake of 11.3% (95% CI, 8.9–14.1%) (Figure 7 and Table 1).

An exploratory unadjusted comparison indicated substantial heterogeneity in apparent bait uptake among the seven region-campaign groups (Pearson’s χ^2^ = 622.75, degrees of freedom (df) = 6, *p* < 0.001). Because not all regions were evaluated during every campaign, this finding was interpreted as heterogeneity among region-campaign groups rather than as evidence of independent regional or seasonal effects.

Within Zhambyl Region, where three campaigns were conducted, apparent uptake differed among spring 2025, autumn 2025 and spring 2026 (χ^2^ = 11.49, df = 2, *p* = 0.003). In Kyzylorda Region, apparent uptake did not differ significantly between autumn 2025 and spring 2026 (χ^2^ = 2.78, df = 1, *p* = 0.095).

During spring 2026, apparent uptake differed among Kyzylorda, Zhambyl and Akmola Regions (χ^2^ = 467.91, df = 2, *p* < 0.001). Using Akmola Region as the reference, the unadjusted odds of apparent bait uptake were higher in Kyzylorda Region (odds ratio (OR), 13.51; 95% CI, 9.99–18.28) and Zhambyl Region (OR, 23.89; 95% CI, 16.60–34.39). The unadjusted odds were also higher in Zhambyl than in Kyzylorda Region (OR, 1.77; 95% CI, 1.30–2.41). Thus, the lowest apparent uptake was observed in Akmola Region during spring 2026, whereas the highest was observed in Zhambyl Region during the same campaign.

The field campaigns were conducted under markedly different temperature conditions. Descriptive mean temperatures across the locality-specific campaign periods ranged from 4.7 to 19.1 °C. The lowest mean temperature was recorded during the Zhanakorgan campaign in November 2025 (4.7 °C), which also included six freeze–thaw days. By contrast, the highest descriptive mean temperature was recorded during the Zhanakorgan campaign in March 2026 (19.1 °C). The Akmola campaign, conducted from 20 April to 5 May 2026, was characterised by a descriptive mean temperature of 10.1 °C, three days with indicated precipitation and no identified freeze–thaw days. Across all campaigns, the number of days with indicated precipitation ranged from zero to five, whereas freeze–thaw conditions were primarily observed during the late-autumn campaign in Zhanakorgan.

Campaign-specific environmental indicators are summarised in Supplementary Materials Table S1. These environmental observations were descriptive, and no causal association between individual meteorological variables and apparent bait uptake was inferred.

### 3.5. Tetracycline Marking and VNA Seropositivity in Free-Ranging Foxes

Ten free-ranging foxes were captured 21 days after manual bait placement, including five animals in Shieli District, Kyzylorda Region, and five in T. Ryskulov District, Zhambyl Region. All animals were clinically active at the time of examination, with no overt neurological or behavioural abnormalities. Tetracycline fluorescence was detected in the dental tissues of seven foxes, corresponding to a marker-positive proportion of 70.0% (95% CI, 34.8–93.3%).

**Figure 8:**
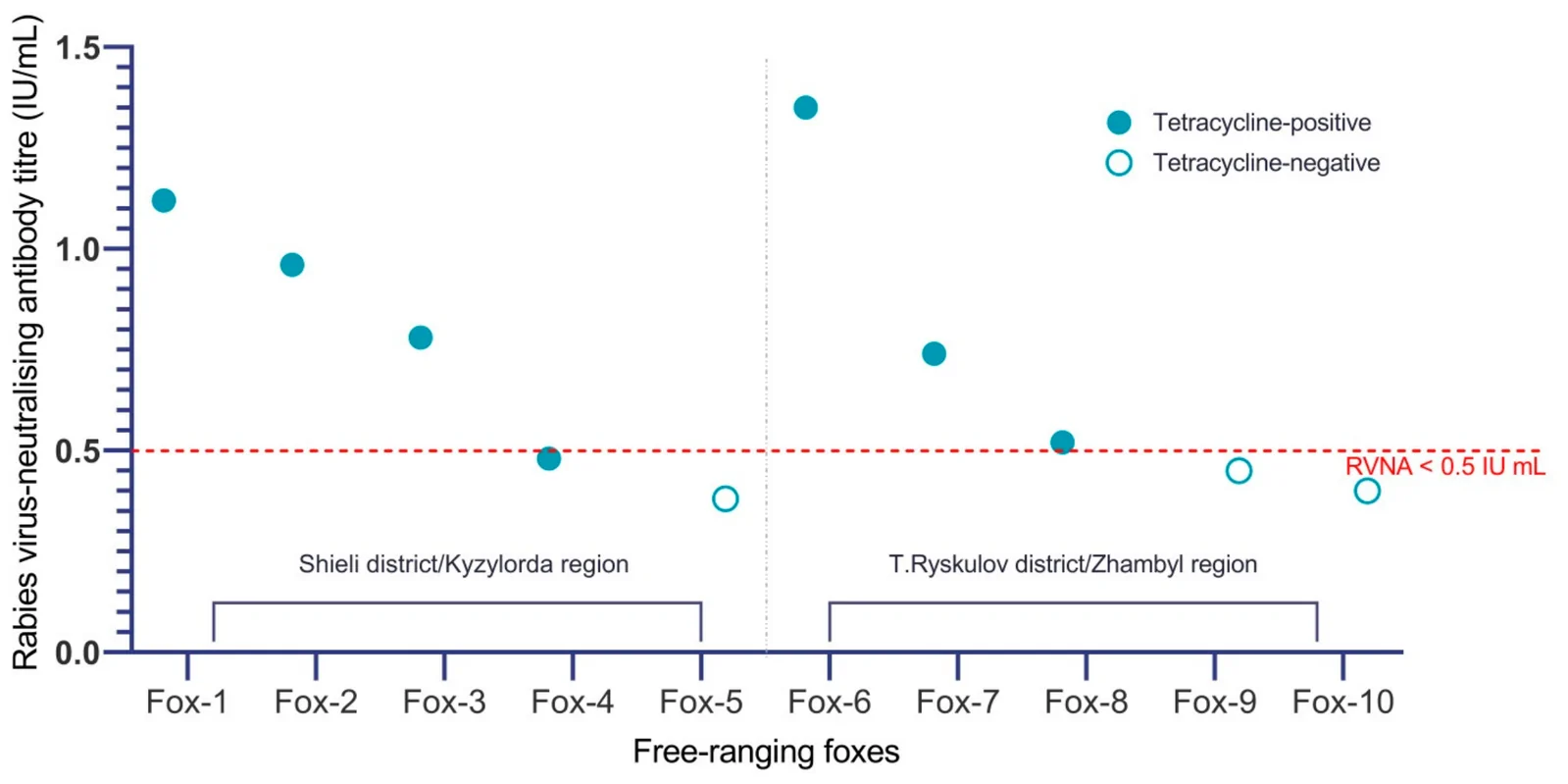
Tetracycline biomarker status and rabies virus-neutralising antibody titres in free-ranging foxes after field bait deployment. Individual points show RVNA titres in ten free-ranging foxes sampled 21 days after manual bait placement in Shieli District, Kyzylorda Region (*n* = 5), and T. Ryskulov District, Zhambyl Region (*n* = 5). Filled symbols indicate foxes with detectable tetracycline fluorescence, whereas open symbols indicate foxes without detectable fluorescence. The horizontal dashed line indicates the prespecified RVNA seropositivity threshold of 0.5 IU/mL.

Rabies virus-neutralising antibody (RVNA) titres of at least 0.5 IU/mL were detected in six foxes (60.0%; 95% CI, 26.2–87.8%). Among RVNA-seropositive animals, individual titres ranged from 0.52 to 1.35 IU/mL, whereas the remaining four foxes had titres below 0.5 IU/mL (Figure 8).

Six of the seven foxes with detectable tetracycline fluorescence had RVNA titres ≥ 0.5 IU/mL (85.7%), compared with none of the three foxes without detectable fluorescence. The association between tetracycline biomarker detection and RVNA seropositivity was statistically significant in a two-sided Fisher’s exact test (*p* = 0.033). However, this result should be interpreted cautiously because of the small sample size and the inclusion of animals from two capture locations.

Because pre-deployment serum samples were unavailable, these findings were interpreted as exploratory post-campaign evidence of an association between tetracycline-marked bait exposure and RVNA seropositivity, rather than as confirmation of individual seroconversion or protection against rabies.

### 3.6. A Single Oral Bait Induced VNA Seroconversion in Captive Foxes and Raccoons

Controlled oral administration was evaluated in six captive red foxes and five captive raccoons. Before vaccination, all 11 animals were RVNA-seronegative, with no detectable rabies virus-neutralising antibodies.

In red foxes, RVNA titres increased from undetectable levels before vaccination to a median of 0.79 IU/mL on day 21 (interquartile range (IQR), 0.67–1.05 IU/mL). This increase was statistically significant according to an exact two-sided Wilcoxon matched-pairs signed-rank test (*p* = 0.031). All six foxes met the prespecified seroconversion criterion of RVNA ≥ 0.5 IU/mL, corresponding to a seroconversion proportion of 100% (95% confidence interval (CI), 54.1–100%).

In raccoons, RVNA titres increased from undetectable levels before vaccination to a median of 0.85 IU/mL on day 21 (IQR, 0.78–0.95 IU/mL). Four of five raccoons met the seroconversion criterion, corresponding to a seroconversion proportion of 80.0% (95% CI, 28.4–99.5%). The paired increase did not reach statistical significance according to an exact two-sided Wilcoxon matched-pairs signed-rank test (*p* = 0.125), most likely reflecting the small sample size. One raccoon had no detectable RVNA response on day 21. This animal carried the bait into its shelter shortly after administration, preventing continuous visual observation. The bait was not subsequently recovered, and complete rupture of the vaccine sachet could therefore not be confirmed.

**Figure 9:**
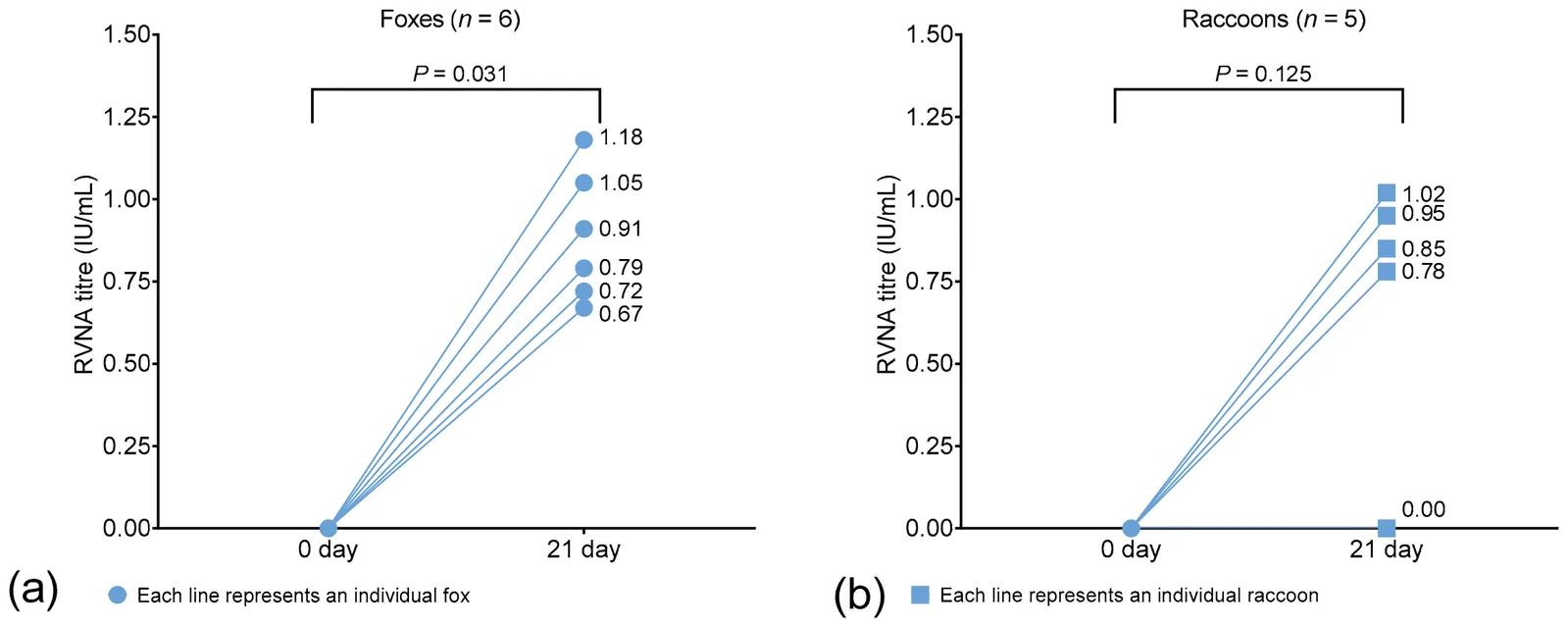
Rabies virus-neutralising antibody (RVNA) titres in captive red foxes and raccoons before and after oral vaccination. (**a**) Red foxes (*n* = 6). (**b**) Raccoons (*n* = 5). Each line represents an individual animal, showing RVNA titres before vaccination (day 0) and 21 days after oral administration of a single briquette-formulated rabies vaccine bait. All red foxes and four of five raccoons reached the predefined seroconversion threshold (RVNA ≥ 0.5 IU/mL) on day 21. Exact two-sided Wilcoxon matched-pairs signed-rank test: foxes, *p* = 0.031; raccoons, *p* = 0.125.

Overall, 10 of 11 initially seronegative animals met the RVNA seroconversion criterion following a single oral administration, corresponding to an overall seroconversion proportion of 90.9% (95% CI, 58.7–99.8%). In an exploratory pooled analysis, RVNA titres increased significantly from pre-vaccination levels to day 21 (exact two-sided Wilcoxon matched-pairs signed-rank test, *p* = 0.002). However, species-specific outcomes were considered primary because red foxes and raccoons are biologically distinct (Figure 9).

### 3.7. Exploratory Observations in Non-Target Animals and During Field Handling

Five captive bears were included in an exploratory assessment of bait acceptance and short-term clinical tolerance. Despite the availability of their routine food (fresh fish) in the enclosure, all bears readily consumed the vaccine baits, indicating good bait acceptance under the conditions of the study. Each animal consumed ten vaccine baits. No visible neurological abnormalities, behavioural changes, reduced appetite or other overt clinical abnormalities were recorded during the 35-day observation period. Blood samples were not collected because sampling would have required sedation; consequently, no serological outcomes were available for this species.

During field deployment and subsequent site monitoring, no accidental human exposure to vaccine suspension was recorded. No visible leakage from vaccine sachets was identified during routine field handling, and no personnel injuries or other vaccine-related operational incidents were documented. These observations were descriptive and should not be interpreted as a formal safety assessment.

## 4. Discussion

In this pilot multi-region field implementation study, the overall apparent uptake of manually monitored oral rabies vaccine baits reached 57.2%, demonstrating the operational feasibility of the briquette-formulated bait under contrasting ecological conditions in Kazakhstan. However, bait uptake varied markedly among region-campaign groups, ranging from 11.3% to 75.3%, indicating that local ecological and operational conditions strongly influenced field performance. Tetracycline biomarker detection in free-ranging foxes and RVNA responses in both free-ranging and captive animals further supported the biological activity and immunogenic potential of the vaccine following oral administration. Together, these findings support our hypothesis that region-specific ecological characteristics should be considered when designing oral rabies vaccination programmes rather than applying a uniform bait-distribution strategy across contrasting landscapes.

A total of 9256 vaccine baits were deployed during three field campaigns, including 4556 placed manually and 4700 distributed by aircraft. Kazakhstan is characterised by vast territories and heterogeneous landscapes that present logistical challenges for wildlife rabies control. Under these conditions, aerial distribution offers broader coverage of remote areas, whereas manual placement allows targeted bait deployment in microhabitats associated with wild carnivore activity, such as animal trails, dens, ravines and water sources. Similar combinations of manual and aerial bait distribution have been successfully implemented in wildlife oral rabies vaccination programmes in Europe and North America [7,19].

All 78 baits recovered after the controlled aerial drop test retained their external shape and contained intact vaccine sachets without visible leakage, demonstrating the mechanical suitability of the briquette formulation for aerial deployment under the tested conditions. Nevertheless, because 22 baits were not recovered, their post-release condition could not be assessed.

Overall apparent uptake among manually monitored baits was 57.2%, ranging from 11.3% in Akmola Region to 75.3% in Zhambyl Region during spring 2026; uptake in Kyzylorda Region during the same campaign was 63.3%. Camera traps frequently recorded red foxes and golden jackals at southern bait sites, whereas corsac foxes were the principal target canids detected in Akmola Region. These differences may reflect variation in local species composition, animal activity, habitat characteristics and bait placement. However, apparent uptake included both complete consumption and partial damage, and therefore did not confirm sachet rupture or successful delivery of the vaccine suspension.

The marked variation among region-campaign groups cannot be attributed to a single environmental or biological factor. It may reflect differences in target-canid activity, species-specific feeding behaviour, alternative food availability, vegetation structure, bait visibility, microhabitat selection and competition from non-target animals. Because the study sites were purposively selected and wildlife abundance was not standardised across locations, the low apparent uptake observed in Akmola Region should not be interpreted as evidence that the bait formulation is unsuitable for northern or steppe landscapes more broadly. Rather, these findings highlight the importance of regional ecological context when planning oral rabies vaccination campaigns. In Akmola Region, where the study was conducted in open steppe habitats, corsac foxes were the principal target canids recorded by camera traps. Differences in habitat structure, reservoir-species composition and animal movement patterns may therefore have influenced bait encounter rates compared with the more heterogeneous southern study areas. These observations suggest that future studies should evaluate whether region-specific adjustments in bait density, spatial placement, deployment timing and microhabitat selection could improve bait accessibility and uptake under steppe conditions. Overall, these findings underscore the importance of an adaptive, region-specific approach to oral rabies vaccination rather than the uniform application of a single bait-distribution strategy across ecologically contrasting landscapes.

Although rabies prevalence is not consistently related to reservoir-host density [20], ORV effectiveness depends partly on the spatial distribution and activity of reservoir species and on the proportion of animals that encounter and consume vaccine baits [21]. International experience shows that vaccination coverage may be reduced when animal activity is spatially heterogeneous or when bait density and distribution do not correspond to local habitat use [19,22,23]. The present findings therefore support locally adapted bait densities, campaign timing and placement strategies rather than the uniform application of a single distribution scheme across contrasting landscapes.

The field campaigns were conducted under different meteorological conditions, with mean temperatures ranging from 4.7 to 19.1 °C and variation in precipitation and freeze–thaw events. These conditions may have influenced bait integrity, animal activity and food availability; however, the study was not designed to estimate the independent effects of individual meteorological variables. Environmental data should therefore be interpreted as descriptive campaign characteristics rather than evidence of causal associations with apparent bait uptake.

Camera-trap detections occurred mainly during the evening, night and early morning, consistent with the predominantly nocturnal or crepuscular activity of wild canids. Recorded behaviours included bait investigation, partial or complete consumption, displacement and removal. Non-target species, including porcupines, badgers, wild boars and birds, were also detected. Similar studies in South Africa and North America have shown that bait disappearance may result from interactions by both target and non-target species, and therefore does not necessarily indicate vaccination of the intended reservoir population [24,25]. Likewise, field studies in Finland have demonstrated that biomarker detection may exceed seroconversion rates because animals may consume the bait matrix without rupturing the vaccine sachet or may lose part of the vaccine suspension during bait handling [26]. Consequently, apparent bait uptake should be interpreted as an operational indicator of bait accessibility rather than direct evidence of successful vaccine delivery. Combining camera-trap monitoring with physical bait inspection enabled species identification and better differentiation among consumption, investigation and displacement.

Non-target interactions may have reduced bait availability to wild canids, although cameras were installed at only a subset of deployment sites and species-specific removal rates could not be estimated across the study area. Future field campaigns should assess whether bait placement time, concealment, density and microhabitat selection can improve access by target species while limiting non-target removal.

Tetracycline fluorescence was detected in seven of ten free-ranging foxes, and six had RVNA titres ≥ 0.5 IU/mL. Six of the seven tetracycline-positive foxes were RVNA-seropositive, whereas none of the three marker-negative animals reached this threshold. These findings were consistent with an association between exposure to a tetracycline-containing bait and detectable humoral immunity, although the small sample size and absence of baseline sera precluded confirmation of campaign-induced seroconversion.

Differences between bait-marker detection and rabies antibody prevalence have also been reported in other ORV programmes. In Israel, tetracycline markers were detected in approximately 65% of sampled red foxes and 56% of golden jackals [23]. In Finland, markers were detected in approximately three-quarters of sampled foxes and raccoon dogs, whereas antibodies were found in slightly more than half of the animals [26]. Large-scale campaigns in Türkiye likewise demonstrated reductions in fox-mediated rabies despite incomplete marker detection and moderate post-vaccination seroprevalence [27]. The tetracycline-positive proportion observed in this study was broadly consistent with these reports, although direct comparisons should be interpreted cautiously because of differences in bait formulation, target species, sampling design, campaign conditions and laboratory methods. Tetracycline marking and RVNA testing provided complementary rather than equivalent measures of vaccine exposure: the biomarker indicated previous bait consumption, whereas RVNA reflected a measurable humoral immune response following successful vaccine delivery. However, only ten free-ranging foxes from two locations were examined, baseline sera were unavailable and confidence intervals were wide. Therefore, these findings provide exploratory evidence of an association between bait exposure and RVNA seropositivity rather than definitive evidence of campaign-induced seroconversion or long-term protective immunity. The present study was not designed to evaluate the duration of immunity or protective efficacy following field exposure. Consequently, the observed RVNA titres should be interpreted as evidence of vaccine-induced humoral responses rather than proof of long-term protection. Evaluation of the persistence and protective significance of these antibody responses will require longitudinal serological follow-up and, where appropriate, controlled challenge studies.

Controlled oral administration provided stronger evidence of immunogenicity. Following administration of a single bait, all six captive red foxes and four of five captive raccoons reached the predefined RVNA threshold. Because all animals were seronegative before administration, these results demonstrate that the bait delivered biologically active vaccine and induced a measurable humoral response under controlled conditions. Comparable studies with other oral rabies vaccines have also reported substantial antibody responses in raccoons, although response magnitude may vary with vaccine type, bait formulation and deployment conditions [28].

The absence of a detectable RVNA response in one raccoon should be interpreted with caution. Shortly after bait administration, this animal carried the bait into its shelter, preventing continuous visual observation. The bait was not subsequently recovered by either the investigators or zoo personnel, and complete rupture of the vaccine sachet could therefore not be confirmed. Consequently, incomplete vaccine exposure cannot be excluded as a possible explanation for the absence of seroconversion. However, the small sample size precluded meaningful comparisons between species. Raccoons do not occur naturally in Kazakhstan and were included only as an additional captive species for assessing bait acceptance and serological response.

Five captive brown bears were included solely to document bait acceptance and short-term clinical tolerance following oral administration of the vaccine bait. Despite the simultaneous availability of their routine food, all bears readily consumed the vaccine baits, indicating good palatability of the bait formulation for this non-target species. No clinical abnormalities were observed during the 35-day monitoring period. However, these observations should be interpreted as evidence of short-term clinical tolerance only. No investigations of viral replication, viral shedding, tissue distribution or post-mortem pathology were performed. Therefore, the present findings do not permit conclusions regarding vaccine safety in bears and should be regarded as preliminary observations requiring dedicated safety studies.

A major strength of this pilot study was the integration of GPS-referenced deployment, assessment of post-release bait integrity, camera-trap monitoring, tetracycline biomarker detection and RVNA testing. Together, these approaches evaluated several sequential components of ORV implementation and extended previous laboratory and preclinical assessments of the formulation [15,16] to operational field conditions across selected landscapes.

This study also had important limitations. Study districts were purposively selected, the number of free-ranging foxes available for biomarker and serological assessment was small, and baseline sera were unavailable. Camera traps covered only a subset of deployment sites, and apparent bait uptake did not confirm successful vaccine delivery for every monitored bait. The controlled oral administration groups were also small and were intended to provide supporting evidence of immunogenicity and clinical tolerability rather than definitive interspecies comparisons. Furthermore, this study was not designed to assess nationwide effectiveness, changes in rabies incidence, interruption of transmission, long-term immunity or programme-level cost-effectiveness.

Overall, the findings support further evaluation of the briquette-formulated oral rabies vaccine bait under the ecological conditions represented by the selected study sites. Relatively high apparent uptake at several southern sites, preservation of bait integrity after controlled aerial release, tetracycline detection in free-ranging foxes and RVNA responses after oral administration provided complementary evidence of operational feasibility and biological activity. The substantial variation among region-campaign groups indicates that bait density, campaign timing, placement method and monitoring should be adapted to local environmental conditions and reservoir-species ecology. These results provide a basis for larger field studies incorporating broader sampling of free-ranging canids, site-level analyses and longitudinal rabies-surveillance outcomes.

## 5. Conclusions

This pilot multi-region study provides integrated biological and operational evidence supporting the field applicability of a locally developed briquette-formulated oral rabies vaccine bait in Kazakhstan. The formulation retained its physical integrity under the tested aerial release conditions, was accessed by free-ranging target canids across contrasting landscapes, and elicited rabies virus-neutralising antibody seroconversion in all six initially seronegative captive red foxes and four of five raccoons following a single observed oral administration. In free-ranging foxes, the close overlap between tetracycline biomarker detection and RVNA seropositivity provided complementary exploratory evidence linking marked bait exposure with detectable humoral immunity.

The substantial variation in apparent bait uptake among region-campaign groups demonstrates that successful field performance depends not only on bait formulation but also on local reservoir-species composition, animal activity, habitat characteristics and deployment strategy. These findings support the use of ecologically adapted bait densities, placement methods and campaign timing rather than uniform distribution approaches across contrasting landscapes. Although the present results do not establish nationwide effectiveness, protective immunity in free-ranging animals or interruption of rabies transmission, they provide a strong biological and operational basis for larger controlled field studies incorporating site-level analyses, broader sampling of target canids, longitudinal serological monitoring and rabies-surveillance outcomes.

## Figures and Tables

**Table 1:** Field campaign characteristics and apparent oral rabies vaccine bait uptake.

Campaign	Region	Study Districts	Field Period	Deployment Method	Baits Deployed	Baits Monitored	Consumed or Damaged, n	Intact, n	Apparent Uptake, % (Exact 95% CI)
Spring 2025	Turkestan	Otyrar and Kazygurt	19–31 March 2025	Manual	740	740	459	281	62.0 (58.4–65.5)
	Zhambyl	T. Ryskulov and Baizak	1–16 April 2025	Manual	740	740	502	238	67.8 (64.3–71.2)
Autumn 2025	Kyzylorda	Shieli and Zhanakorgan	25 October–11 November 2025	Manual	856	856	505	351	59.0 (55.6–62.3)
	Zhambyl	T. Ryskulov and Merke	12–27 November 2025	Manual	720	720	464	256	64.4 (60.8–67.9)
Spring 2026	Kyzylorda	Shieli and Zhanakorgan	17–31 March 2026	Manual	600	600	380	220	63.3 (59.3–67.2)
	Zhambyl	T. Ryskulov	1–10 April 2026	Manual	300	300	226	74	75.3 (70.1–80.1)
	Akmola	Astrakhan	20 April–5 May 2026	Manual	600	600	68	532	11.3 (8.9–14.1)
	Zhambyl	T. Ryskulov, Merke, Bayzak	1–10 April 2026	Aerial	4700	Not assessed	Not assessed	Not assessed	Not assessed
Manual deployment total	-	-	-	Manual	4556	4556	2604	1952	57.2 (55.7–58.6)
Overall deployment total	-	-	-	Manual and aerial	9256	-	-	-	-

## Data Availability

All data supporting the findings of this study are included in the article. The raw data are available from the corresponding author upon reasonable request.

## Supplementary Materials

The following supporting information can be downloaded at: https://www.mdpi.com/article/10.3390/biology15161374/s1, Table S1: Regional climatic context of field bait deployment campaigns.

## Abbreviations

BHK-21	Baby hamster kidney-21
BSL-3	Biosafety level 3
CI	Confidence interval
CVS	Challenge virus standard
DMEM	Dulbecco’s modified Eagle medium
FBS	Fetal bovine serum
FITC	Fluorescein isothiocyanate
GPS	Global positioning system
IQR	Interquartile range
OR	Odds ratio
ORV	Oral rabies vaccination
PM1	First premolar tooth
RFFIT	Rapid fluorescent focus inhibition test
RIBSP	Research Institute for Biological Safety Problems
RVNA	Rabies virus-neutralising antibodies
TCID_50_	Median tissue culture infectious dose
UV	Ultraviolet
