# Supplementary material for: Experimental Immunogenicity and Multi-Region Field Performance of a Briquette-Formulated Oral Rabies Vaccine Bait in Kazakhstan
Source: Biology (Basel). 2026 Aug 12;15(16):1374. doi: 10.3390/biology15161374 (PMC13509442; doi:10.3390/biology15161374)
Supplement: Supplementary file 1 [file biology-15-01374-s001.zip › biology-4488152-supplementary.pdf]

Table S1. Regional climatic context of field bait deployment campaigns

Campaign	Region	Period	Reported monthly temperature range, °C	Precipitation context	Important limitation
Spring 2025	Turkestan	March 2025	+3.3 to +12.1	Near normal; above normal in southeast	Regional forecast, not observed site-level data
Spring 2025	Zhambyl	March 2025	+3.2 to +7.8	Above normal across most areas	Regional forecast
Spring 2025	Turkestan	April 2025	+6.9 to +16.9	Near normal; higher in mountains and foothills	Regional forecast
Spring 2025	Zhambyl	April 2025	+10.3 to +14.1	Near normal; higher in mountains and foothills	Regional forecast
Autumn 2025	Zhambyl	October 2025	+8.4 to +12.8	Near normal; higher in southern areas	Regional forecast
Autumn 2025	Kyzylorda	October 2025	+9.9 to +12.7	Near normal	Regional forecast
Spring 2026	Akmola	20 Apr–10 May 2026	Not suitable for quantitative reporting from current screenshots	Calendar displays climatological averages	Ten-year averaged data, not observations

Campaign	Region and locality	Exposure period	Days	Mean Tmax, °C	Mean Tmin, °C	Mean temperature, °C	Days with indicated precipitation	Freeze–thaw days	Data source
Spring 2025	Otyrar District, Turkestan Region	19–24 Mar 2025	6	24.5	12.0	18.2	1	0	Kazygurt March 2025 calendar used as a regional proxy[1]
Spring 2025	Kazygurt District, Turkestan Region	24–31 Mar 2025	8	13.2	5.6	9.4	5	0	Kazygurt monthly weather calendar
Spring 2025	Ryskulov District, Zhambyl Region	1–6 Apr 2025	6	20.0	7.5	13.8	3	0	Ryskulov monthly weather calendar
Spring 2025	Ryskulov District, Zhambyl Region	7–16 Apr 2025	10	21.9	10.2	16.0	2	0	Ryskulov monthly weather calendar
Autumn 2025	Shieli District, Kyzylorda Region	25–31 Oct 2025	7	17.4	6.0	11.7	0	0	Shieli monthly weather calendar
Autumn 2025	Zhanakorgan District, Kyzylorda Region	1–11 Nov 2025	11	10.2	−0.7	4.7	1	6	Zhanakorgan monthly weather calendar
Autumn 2025	Ryskulov District, Zhambyl Region	12–19 Nov 2025	8	15.4	3.4	9.4	0	0	Ryskulov monthly weather calendar
Autumn 2025	Merke District, Zhambyl Region	20–27 Nov 2025	8	12.8	1.2	7.0	0	0	Merke monthly weather calendar
Spring	Shieli	17–25	9	19.6	7.2	13.4	0	0	Shieli

2026	District, Kyzylorda Region	Mar 2026							monthly weather calendar[2]
Spring 2026	Zhanakorgan District, Kyzylorda Region	25–31 Mar 2026	7	26.6	11.7	19.1	0	0	Zhanakorgan monthly weather calendar[2]
Spring 2026	Kulan, T. Ryskulov District, Zhambyl Region	1–10 Apr 2026	10	19.9	10.3	15.1	4	0	Kulan monthly weather calendar[2]
Spring 2026	Astrakhanka, Astrakhan District, Akmola Region	20 Apr–5 May 2026	16	15.9	4.3	10.1	3	0	Yandex monthly calendar; long-term averaged conditions[3]
